# Cerebral blood flow and arterial responses in migraine: history and future perspectives

**DOI:** 10.1186/s10194-024-01903-2

**Published:** 2024-12-19

**Authors:** Jes Olesen

**Affiliations:** Translational Research Center and Danish Headache Center, Rigshospitalet, University of Copenhagen, Nordstjernevej 42, Glostrup, Copenhagen, 2600 Denmark

## Abstract

**Introduction:**

It is largely accepted that migraine with aura (MA) is caused by cortical spreading depression (CSD) and that migraine without aura (MO) is not. This is mostly based on old studies of regional cerebral blood flow (rCBF) and studies of vascular responses. These studies are partly forgotten today and may, therefore, be worthwhile reviewing.

**Methods:**

The review is based on the authors life-long involvement in these issues and his knowledge of the relevant literature plus scrutiny of reference lists of these papers.

**Results:**

The strongest evidence for CSD in MA came from studies using intraarterial injection of 133-Xenon and recording from 254 areas of the relevant hemisphere. Measurements could be taken before and during development of an attack because the procedure triggered MA. The findings were identical to many features of CSD. They were confirmed using 133-Xenon Single Photon Emission Computerized Tomography (SPECT).It was shown that the generally accepted vasospastic theory of migraine was incorrect. Headache started while rCBF was decreased and did not change during later hyperperfusion. rCBF remained normal in MO but later studies have shown increase in areas also activated by other pain. Flow Was focally increased in the brain stem also after treatment of the pain. Dilatation of large cerebral arteries during MO attack was first shown with ultrasound and later confirmed by MR angiography which also showed a lack of dilatation of extracerebral arteries.

**Discussion:**

Much has in later years been done using modern PET and MR techniques. These studies have confirmed the old studies and have added many new aspects which are not reviewed here. The final proof of CSD during MA and its absence during MO still awaits the definitive study.

**Conclusion:**

Studies from the 1980ies and 1990ies caused a fundamental shift in our understanding of the vascular and cortical mechanisms of migraine. They remain a solid base for our current understanding and inspire further study.

## Introduction

Is migraine a vascular- or a brain disorder? This question has been the subject of enormous interest during more than a century with some adamantly supporting a “vascular theory” and others equally strongly supporting that migraine is a disorder of the central nervous system (CNS) [1]. Wollf`s monumental research two generations ago strongly emphasized the vascular theory, and this view dominated the last half of the 20ies century [2]. But then the tide turned, and migraine was viewed as a brain disorder while vascular involvement was considered an unimportant epiphenomenon. The present author has always defended a middle way proposing that migraine is a neuro-vascular disorder [3]. It acknowledges that migraine has initiating brain mechanisms but also, that pain signaling is generated by the perivascular innervation of cephalic blood vessels. In other words, the nociception of migraine originates from trigeminal perivascular sensory nerve fibers [3]. This view has recently gained momentum because of new effective drugs that hardly cross the blood-brain barrier (BBB) and because of migraine induction by peptides that also do not cross.

Much of the evidence for vascular changes during migraine was obtained many years ago and may have been forgotten by present day headache experts. It is for them that this review is written. It is a last witness account by a scientist who has significantly contributed to the early study of brain blood flow and arterial reactions, studies that have changed the way in which we look at migraine. The very productive period in the 1980ies and 1990ies is comprehensively discussed and later studies have been selectively included. Most of these old studies have not been repeated and the studies of regional cerebral blood flow (rCBF) in migraine with aura (MA) patients will probably never be repeated. They were only possible because carotid arteriography triggered the attacks. This investigation is not used anymore. A detailed account of these studies is therefore important today.

The early studies showed for the first time a marked differences between MA and migraine without aura (MO). The rCBF studies strongly suggested that MA was the clinical manifestation of cortical spreading depression (CSD) [4] while such changes were not found in migraine without aura (MO) [5]. These differences strongly supported that the two subtypes of migraine be diagnostically clearly distinguished. The rCBF findings in MA were the main reason why the International Classification of Headache Disorders (ICHD-1) [6] provided separate explicit diagnostic criteria for MA and MO. They have also resulted in valid animal models of MA and novel drug development There is no evidence of CSD in MO, but a lot is known about its arterial reactions and causative signaling mechanisms from the early studies. All this is also discussed. Finally, a personal view of the possibilities offered by further studies of brain blood flow and arterial reactions is presented.

Better and better methods have become available for the measurement of brain function such as various modalities of MR and PET. They have yielded a wealth of information about possible changes during- and outside of migraine attack. But the results have often been inconsistent and difficult to replicate. They will not be discussed in this review.

## Methods

The present review is based on original publications and reviews by the author and on the literature lists of such publications. No formal PubMed or other search was performed.

## Results

### Wolff`s vasospastic theory and early CFB studies

During the first half of the twenties century, it was widely believed that migraine pain had a vascular origin. This was based on several simple observations: The pulsating nature of the pain, the headache caused by vascular brain disease and by vasodilators and the therapeutic effect of vasoconstrictors such as ergotamine. Building on this Wolff ingeniously used available simple techniques and showed increased extracranial pulsations during migraine attack. Cerebral blood vessels did not cause the pain as intrathecal saline infusion to raise intracranial pressure did not affect the pain. He realized that the simple mechanical force of the modest dilatation would not be enough to fully explain the pain and hypothesized the leaking of a “headache stuff” around extracranial arteries [2]. In the general understanding and in textbooks the latter aspects were, however, forgotten. It became “common textbook knowledge” that migraine is due to spastic contraction of brain arteries that caused the aura symptoms followed by vasodilatation and increased blood flow causing the pain.

The first quantitative measurements of CBF seemed to confirm this. O`Brien used 133-Xenon (Xe) inhalation and one detector for each hemisphere [7]. He found no difference between hemispheres but decreased CBF during the aura and increased CBF during headache. Skinhøj and Paulson measured from 16 regions of a hemisphere and found rCBF to be decreased in the relevant area [7]. Skinhøj also found increased spinal fluid lactate suggesting ischemia [8]. The results were interpreted as supporting the vasospastic theory. Norris et al. [9] found such evidence in a single case and Mathew et al. [10] using the unprecise method of Xenon inhalation with stationary detectors found decreased flow during so called prodrome and increased flow during headache disregarding migraine type. In a meeting report Hachinski reported 5 cases studied during attack but results were varying [11] Finally, Hachinski et al. [12] using 254 detectors over one hemisphere reported three cases. In so called classic migraine, now migraine with aura, the vasospastic theory was supported. In so called common migraine, now migraine without aura, there was no decreased flow in this single case.

### CBF in migraine with aura

#### Ictal studies

**Fig. 1:**
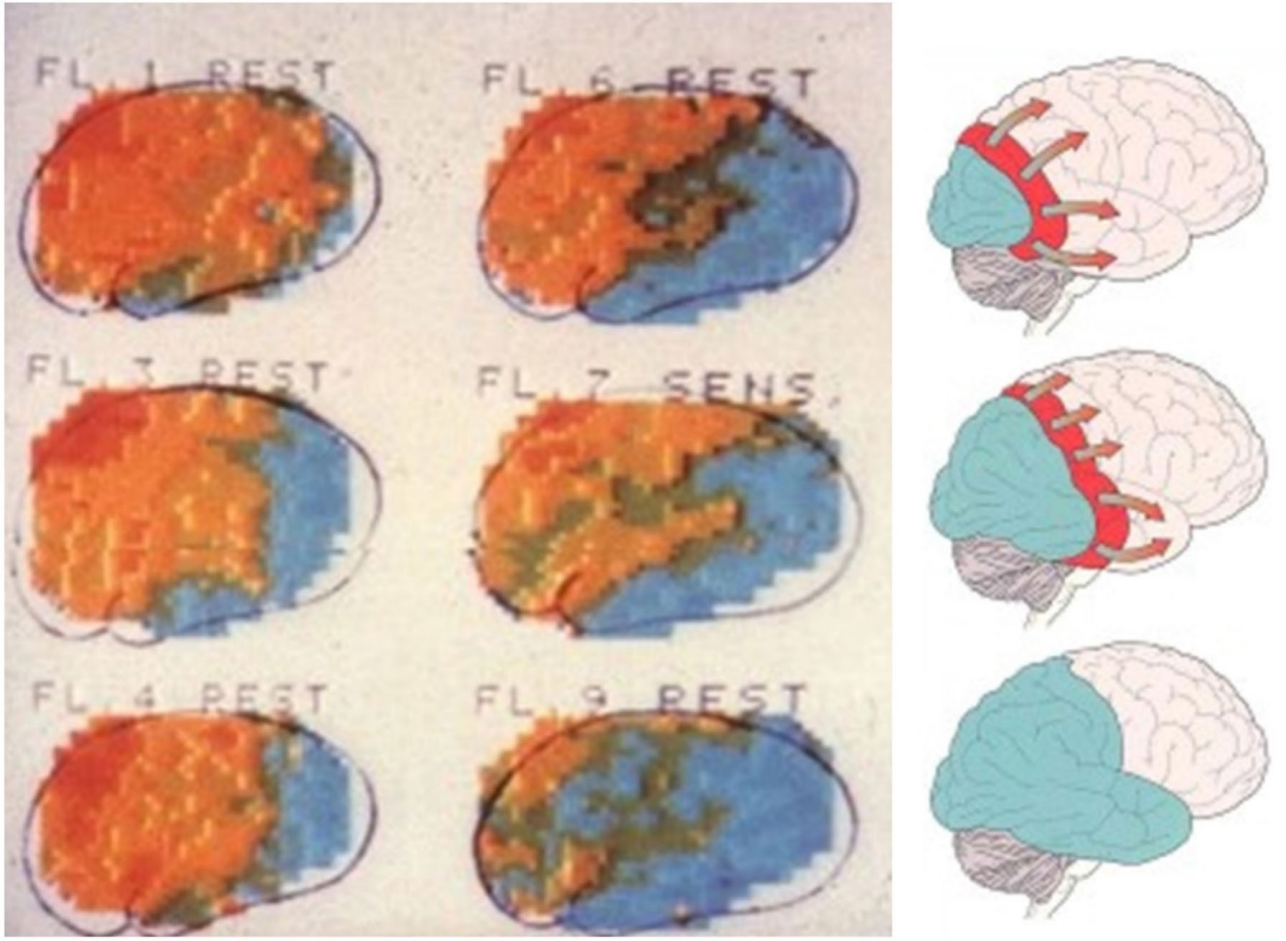
rCBF measured repeatedly during development of an attack of migraine aura. Top left shows a normal flow distribution in the hemisphere but during successive measurements numbered in the figure a blue area at the posterior pole of the hemisphere appears and it expands gradually forward. These changes are very similar to changes during CSD shown schematically on the right side of the figure. Adapted from Lauritzen et al. [15]

While using a newly developed equipment with 254 stationary detectors for routine measurement of rCBF in difficult neurological cases, some strange results were obtained in 6 patients. It turned out that their clinical features were compatible with migraine with aura [13]. The results were iconoclastic. There was an initial small are of increased perfusion in three patients followed during sequential measurements by low flow in all 6 patients. It started at the posterior pole of the brain and spread gradually forward for 15–45 min, a speed compatible with the animal experimental phenomenon cortical spreading depression (CSD). The changes did not respect territories of supply of major brain arteries. Functional activation by hand movement or visual stimulation was abolished in the hypo perfused area. It was concluded that the results contrasted with the vasospastic theory. Scrutinizing the literature then showed excellent studies demonstrating that arteriography both by direct arterial puncture and by catheterization triggered attacks of migraine aura symptoms in a third of the patients [14]. That was the reason why it had been possible to follow rCBF from the resting state and during the development of the aura. With this knowledge it became possible to plan studies more precisely (Fig. 1).

Lauritzen et al. measured at shorter time intervals and were able to calculate the speed of spread of hypoperfusion to 2.2 mm but rather 3–4 mm taking cortical convolutions into account [15]. They also studied functional activation by hand movement and by changes in blood pressure and CO_2_ [16]. The former was intact, but the latter decreased. The main conclusion was that the rCBF changes were similar to changes observed during CSD. Later animal experiments showed that there is a prolonged period of low flow after CSD like seen during MA attack [17]. Friberg described three cases of hemiplegic migraine where reduced rCBF started in the frontal region [18]. Analyzing the shape of the clearance curves from the low flow area, he found that they were not smooth. He called it vascular instability. Unfortunately, the phenomenon has not subsequently been confirmed or further studied.

**Fig. 2:**
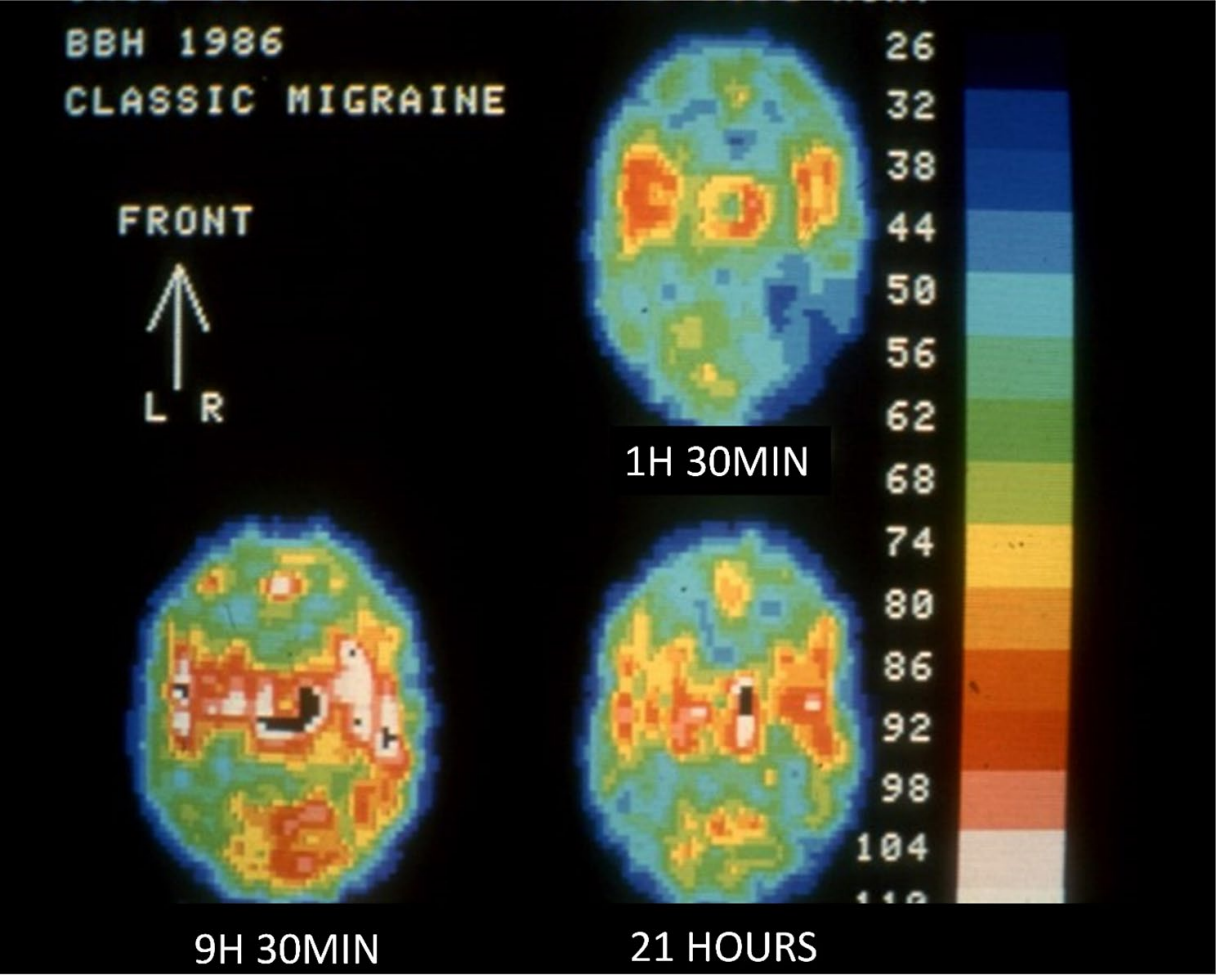
SPECT study of spontaneous attack of migraine with aura. The top picture shows the initial low flow (blue colour) in the right occipital-parietal area. Below to the left the rCBF has changed into hyperperfusion (red and yellow colour). Finally rCBF is normal and symmetrical in the right sided picture. Modified from [21]

Were these provoked attacks the same as spontaneous attacks? A particularly beautiful demonstration of that was provided by fMRi in a patient who could provoke the aura by vigorous physical exercise [19]. The development of single photon computed tomography (SPECT) equipment by Lassen made it possible to obtain rCBF measurements during spontaneous MA attacks. One problem was, however, that spontaneous attacks cannot be studied during their initial development because patients need time to get to the hospital. At the Copenhagen Acute Headache Clinic [20] there was access to patients, some coming for treatment early in the attack. Lauritzen and Olesen [5] demonstrated low flow in the clinically relevant area and could in a few patients even show spread of the low flow area. There was reduced response to CO_2_ changes. In other words, the previous findings during attacks provoked by arteriography were reproduced in spontaneous attacks of MA. SPECT studies with Xenon-133 involve little radioactive irradiation and therefore allow repeated studies. In a seminal study Andersen et al. measured rCBF during- or immediately after the aura and followed patients repeatedly [21]. The well-known low flow early during attack was followed after hours by a period of normal flow and then increased flow in the area of previously low flow. After 24 h or more rCBF was normal (Fig. 2).

**Fig. 3:**
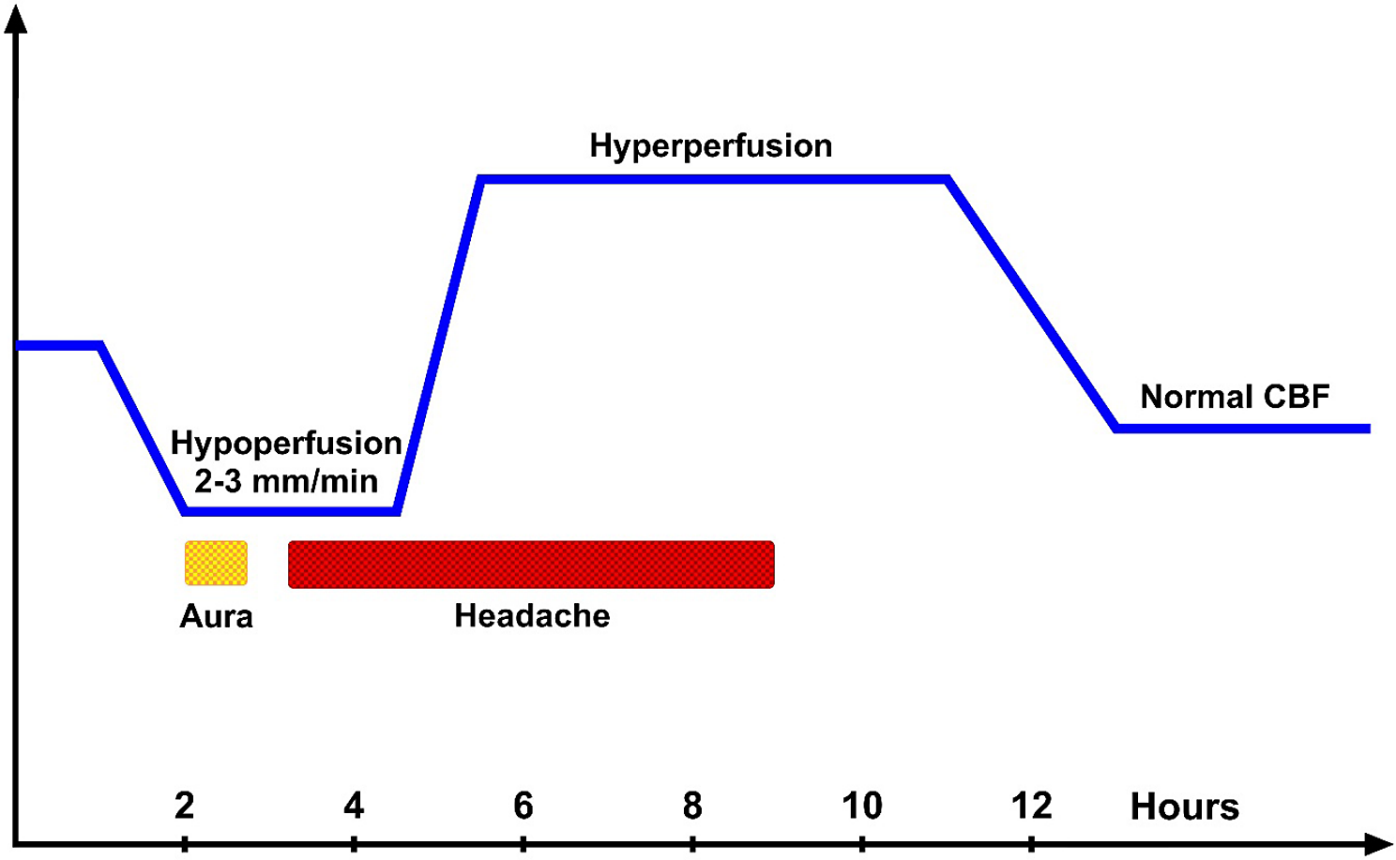
Schematic drawing of events during and attack of MA. Initially rCBF decreases as the aura develops or even slightly before. After the aura has ended low flow continues into the headache phase. During that phase flow changes from low via normal to increased and it remains increased after the headache has disappeared. These changes and several other features ere totally incompatible with the vasospastic theory of migraine but in agreement with CSD as the underlying cause of the attack. Modified from [4]

Olesen et al. combined several previous studies with further unpublished cases in a general analysis of rCBF changes during MA attacks [4] (Fig. 3). An initial low flow area was often seen before the aura symptoms were recorded. The slow spread of low flow was confirmed. The low flow continued for a variable length of time and then changed via normal flow to increased flow. Headache started during low flow and persisted without change during the increased flow. It often disappeared while flow was still increased. The headache was located to the relevant hemisphere in almost all patients, but unilateral flow reduction was often associated with bilateral headache. The findings were incompatible with the vasospastic theory which could now finally be buried with certainty.

#### Is MA the clinical expression of CSD?

As discussed above there are many reasons why CSD is highly likely to be the clinical manifestation of CSD. The slow spread of symptoms and the speed of rCBF changes are the same and identical to the speed of spread of CSD. The impairment of functional activation and other features also show similarity. In addition, a drug that inhibits CSD is effective prophylaxis of MA but not of MO where CSD is not present [22]. But the final proof is missing. No change in direct current (DC) potential typical of CSD has been shown in migraine even though CSD does happen in severely injured human brain [23].

#### Interictal studies

Most of the older studies using intraarterial injection or inhalation of 133-xe showed no abnormalities of flow in the interictal state but a few cases with focal changes were reported in severe cases of what probably today would be hemiplegic migraine. None of these patients had an MR scan to identify possible ischemic damage. Most of these old studies suffered from one or more serious methodological insufficiencies and they will not be reviewed. A relatively large SPECT study that found “patchy” abnormalities but did not use modern standard statistical methods and has never been confirmed [24]. Lauritzen and Olesen found no such changes and interictal rCBF was normal and symmetrical with no evidence of changes in the ictally abnormal area except in one case [5]. Several subsequent PET studies have not described asymmetries or patchy changes in perfusion [25, 26]. On balance it seems that interictal flow in patients with migraine with typical aura is normal while there is possible asymmetry or focally abnormal flow in hemiplegic migraine. However, it is important in such cases to rule out the possibility of previous ischemic damage before attributing findings to the migraine process itself.

### CBF in migraine without aura

#### Ictal studies

**Fig. 4:**
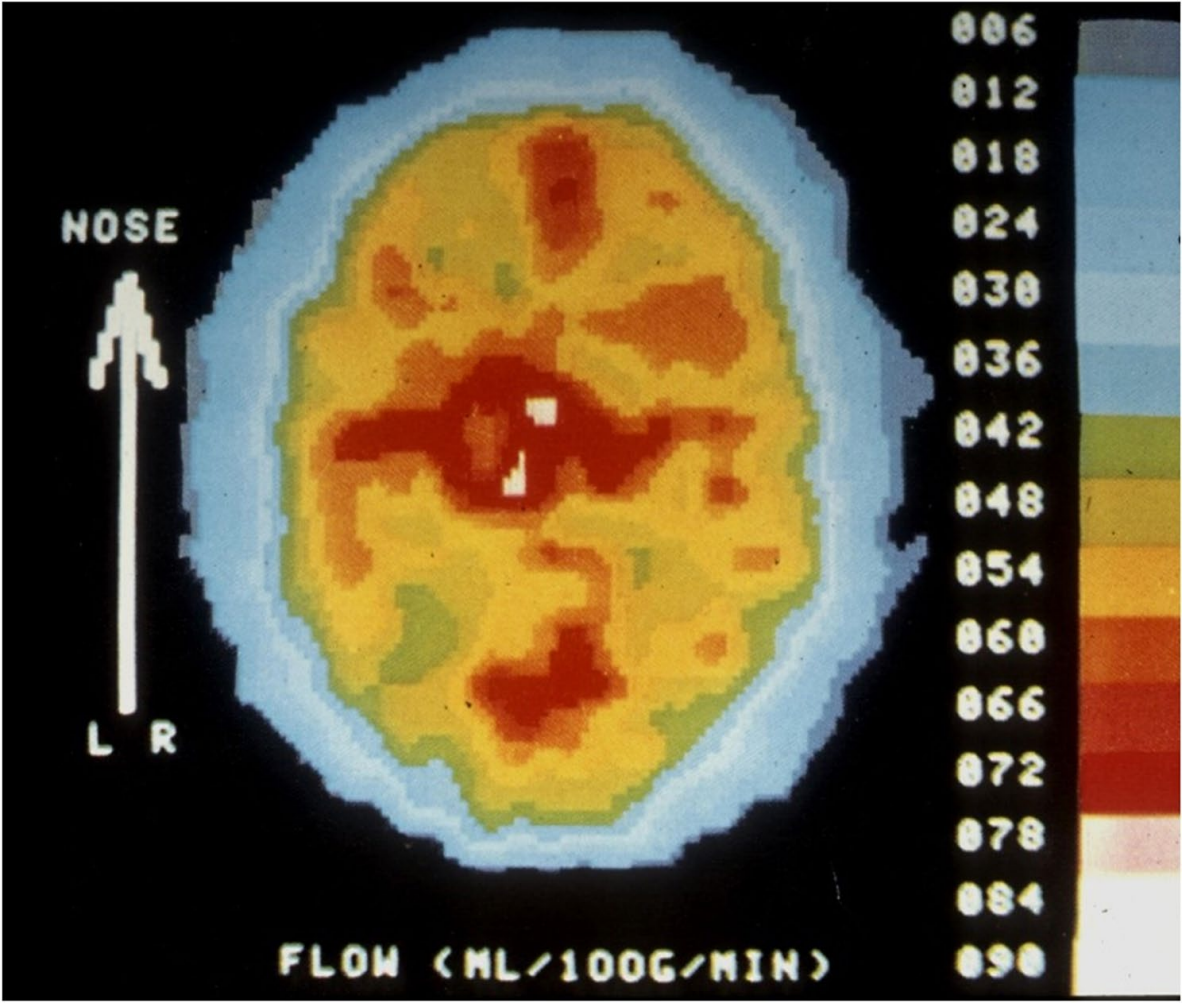
SPECT study of rCBF during an attack of MO. In all such cases rCBF remained normal and symmetrical with the resolution of the technique available that had been able to show focal changes in MA in every case. Subsequent studies with the higher resolution of PET has shown activation in the pain matrix secondary to the pain but nothing that resembles CSD. Modified from [5]

The onset of attack in MA could be measured because carotid angiography, then routinely used for diagnosis, provoked an attack. In MO there was no suspicion of a brain disorder and arteriography was not indicated. rCBF was, however, measured before and after provocation of MO attacks in a few patients who developed migraine shortly after red wine. rCBF was measured with the rather inaccurate 133-Xe inhalation method. No changes were seen during the development of MO attack [27]. Afridi et al. studied attacks induced by glyceryl trinitrate and saw no hemispheric changes resembling CSD although the focus was on brain stem changes [25] All other studies have been done on fully developed attacks. Nevertheless, those studies should have been able to show it if a low flow area as seen in MA were present. It usually persists for hours. Lauritzen and Olesen studied 12 patients with 133-Xe SPECT during attack of MO but found no rCBF abnormalities (Fig. 4) [5].

A large Dutch study used SPECT in patients with MO outside as well as during attack. A normal control group was also included. There were no asymmetries [28]. Weiller et al. in 13 patients studied with PET within 6 h of onset of attack [26] found increased flow only in the so-called pain matrix. It normalized after treatment with sumatriptan and therefore was caused by pain activation. Findings of pain activation were similar in another PET study [25]. In contrast a PET study of seven patients studied within 4 h of onset of MO showed mild posterior hypoperfusion compared to outside of attack [29]. The hypoperfusion persisted after treatment with sumatriptan. It is difficult to explain why these findings differ from those of others cited above. Further study seems warranted, but it is very difficult to do a study shortly after onset of attack. On balance, hemispheric rCBF during attack of MO is most likely normal apart from pain induced activation.

#### Interictal studies

Considering the normal rCBF during MO attack one would of course expect the same to be true outside of attack. That has also been the conclusion in virtually every published study [5, 25, 26, 28, 29]. Missing are again studies prospectively studying sufficiently large patient materials compared to simultaneous normal controls using PET. The trouble of a large and expensive negative study seems to have prevented such studies. They would, however, be worthwhile if functional tests with change of BP, PCO_2_ and hand work were included.

#### Brain stem and hypothalamus

The study of Weiller et al. was valuable because of findings in the hemispheres discussed above. Even more interesting was, however, the demonstration of changes in brain stem blood flow. Seven patients with MO who all had right sided hemicrania were studies within 6 h of onset of attack, then after sumatriptan injection and again headache free after 3–4 months. An 11% increase of flow in brain stem structures not further defined was observed. It persisted when pain free after sumatriptan but was not seen at the migraine free control measurement months later. Because these changes persisted after migraine pain was cured, it was hypothesized that the brain stem activity might somehow drive the migraine attack. Later it has been discussed if it could be called a migraine generator, but the brain stem changes have never been shown to precede the migraine attack. The findings have been confirmed and detailed in attacks provoked by glyceryl trinitrate [25] using a PET equipment with better spatial resolution. Changes could be shown not only in the dorsolateral pons but also in the midbrain. Similar findings were made in 5 cases of spontaneous migraine attack [30]. No changes in the hypothalamus were shown in studies from the 80ies and 90ies possibly due to lack of spatial resolution. A subsequent PET study in 7 patients with MO showed activation in the hypothalamus during attasck [31].

### Controversies and their solution

During the period covered by this review there were three major controversies.

In the early history of rCBF in migraine it was claimed that blood flow was increased during headache even in MO. The publications were mostly from one center, and MO and MA had not been clearly distinguished [32]. It was before ICHD-1. Furthermore, findings were made with techniques that soon after became obsolete. The heated discussions of that time quickly ceased as better diagnosis and measurement techniques showed that there is no hyperemia during MO attacks as discussed above.

Just as it was being accepted based on the studies of Olesen and Lauritzen [13, 16] that migraine aura was the clinical manifestation of CSD, another scientist from Copenhagen by name of Olsen suggested that all previously published studies of rCBF in MA could be explained by cerebral ischemia and so-called Compton scatter, the fact that radiation from healthy areas may influence clearance from low flow areas [33]. It became further chaotic because of the similarity between the Danish surnames Olsen and Olesen. Thus, many thought that the present author had changed opinion about ischemia and CSD. The postulated ischemia was highly unlikely at outset because many patients had aura with minor or undetectable rCBF reduction and for theoretical reasons [34] ubsequent studies with PET and MR showed that tissue is usually not ischemic during aura [19, 35] and the ischemic theory died for the second time after its revival.

As discussed above it is highly likely that spreading hypoperfusion as seen in MA does not happen in MO. Nevertheless, animal experiments have not distinguished between the two types of migraine using CDS models to represent migraine in general [36]. A single case with spontaneous attack of reported MO showed bilateral spreading hypoperfusion [37]. It is the only study showing spreading oligemia in MO patients and the only with bilateral spreading hypoperfusion. Finally, PET studies from one center have shown mildly reduced rCBF in the posterior part of the brain during MO attacks [29]. Posterior flow reduction has not been demonstrated by others with SPECT, PET, or MR as discussed above. On balance, CSD most likely does not underlie MO attacks.

### Arterial reactions in migraine without aura

Given that rCBF is normal during MO, what then is the vascular mechanism of migraine pain? Blood flow is controlled by the arterioles and arteries can dilate or constrict without any change in tissue blood flow. Therefore, normal rCBF does not bespeak what happens in the arteries. In fact, Wolff thought that pain originated from extracerebral arteries [2]. He showed dilatation of the superficial temporal artery but used the uncertain techniques of the time. Decades later high frequency ultrasound scanning of superficial small arteries became available. That showed constriction on the pain free side and unchanged diameter on the pain side during migraine attack [38]. It was hypothesized that pain had activated the sympathetic system to generally constrict arteries but that a local dilatory activity prevented this on the pain side. Wolffs observations were not correct and simple dilatation could not explain the migraine pain. Transcranial dopple (TCD) allowed measurement of velocity of blood in the major basal cerebral arteries. Since rCBF was known to be normal during MO, velocity would be inversely related to the cross sectional area of the arteries. Thomsen et al. [39] measured a 9% decrease in velocity corresponding to a 9% increase of cross-sectional area of the middle cerebral artery. Lots of other TCD studies confirmed or contradicted these results. They will not be reviewed because direct measurement of arterial cross-sectional area with MRA became available as a much better method. Amin et al. [40] managed under incredible difficulties to study 19 patients with MRA during and outside of attack of MO. Pain was half sided in all patients. There was no difference between the painful and the non-painful side of the extracerebral arteries. They also did not difference between attack and outside of attack. In contrast the cerebral arteries on the pain side were dilated compared to the non-painful side and they were also dilated compared to outside of attack. The results of the cerebral arteries were like older studies with ultrasound but added more precision and detail. The findings in the extracerebral arteries differed, however from older studies. Treatment with sumatriptan injection constricted the extracerebral arteries that were normal but not the cerebral arteries that were dilated. It is difficult to explain the findings in a coherent fashion and more studies of the arteries during attack of MO are warranted.

### Vascular origin of migraine pain?

rCBF is largely normal during MO attacks and arterial dilatation is absent outside of the brain and small in the brain. There is good reason to ask the question: Is migraine pain vascular? The answer is still yes for several reasons [3]. The pain often pulsates, and this is worse after physical activity. That is why patients prefer to lie still. GWAS studies have shown 123 variants significantly associated with migraine and they are preferentially expressed in vascular smooth muscle [41]. Headache is a cardinal symptom of a range of vascular brain diseases. Finally, migraine may be induced by several substances and every single one is a vasodilator [42]. In contrast vasoconstrictors like noradrenaline, endothelin, and prostaglandin F2alpha cause no headache at all [43]. The small size of dilatation of arteries during migraine attack is clearly not enough to cause migraine pain by simple stretch of nociceptors. That is why it must be combined with sensitization of afferent nerve fibers. CGRP liberation may do this but seems unlikely to do it alone. Stretch sensitive receptors such as Piezo1 and 2 and TRPM8 may be involved [44]. Probably a whole cascade of biochemical events around cephalic (cerebral and extracerebral) arteries is needed.

### Future possibilities

It is obvious from reading this review that discoveries of rCBF changes during migraine and of arterial reactions has been driven to a large extent by developments in measurement techniques. When better methods for measurement became available, they usually resulted in major steps forward in our understanding. This makes it difficult to predict the future. Therefore, we focus here on some problems that need to be solved and then hope that the technological development will make this possible.

Much of the future progress will not be from the study of spontaneous migraine attacks. It is too difficult to get patients to the hospital, have advanced equipment ready and not taken by another patient. Therefore, attacks must be provoked just like they were by arteriography when that examination was clinically necessary. We know today multiple substances that can induce MO [45]. Such provoked attacks have already been studied but much more needs to be done.

We must be getting close to a final verdict about the migraine aura. Is it or is it not caused by CSD? Years ago, we thought that magnetoencephalography could prove this but it has not happened. Can modern EEG with hundreds of electrodes and advanced IT programs do it? One apparently unsurmountable problem has been the lack of a reliable method for aura provocation. It now seems that some of the MO inducing substances also induce MA in a fraction of patients [46]. That makes study more feasible.

Are we sure that there is no CSD in MO? The French study [29] could suggest that it may be involved but nobody else have seen such flow changes and the results need confirmation.

Modern MR techniques and metabolic and receptor ligand PET offer almost unlimited possibilities for a better understanding of migraine and many studies have already been done. But they are not reviewed in the present paper.

## Conclusion

The older studies of brain blood flow in migraine strongly indicated that the migraine aura is caused by cortical spreading depression. Equally clearly it was shown that such changes do not occur in migraine without aura. This led to a clear distinction between the two subtypes of migraine in the International Classification of Headache Disorders 1st ed and all subsequent editions. The migraine pain was associated with changes in cephalic arteries and sensitization of nociceptors of these arteries combined with mild arterial dilatation remains the best explanation of migraine pain. Much can be learned from the old literature, but even more is to come as techniques for measurement continue to be refined.

## Data Availability

No datasets were generated or analysed during the current study.
